# High KIFC1 expression is associated with poor prognosis in prostate cancer

**DOI:** 10.1007/s12032-021-01494-x

**Published:** 2021-03-24

**Authors:** Laurie G. Kostecka, Athen Olseen, KiChang Kang, Gonzalo Torga, Kenneth J. Pienta, Sarah R. Amend

**Affiliations:** 1 The Brady Urological Institute, Johns Hopkins School of Medicine, 600 N. Wolfe St., Marburg Building Room 113, Baltimore, MD 21287 USA; 2 Cellular and Molecular Medicine Program, Johns Hopkins School of Medicine, 1830 E. Monument St., Baltimore, MD 21205 USA

**Keywords:** Prostate cancer, KIFC1, Centrosome clustering, Ploidy, Kinesins, Poly-aneuploid cancer cells

## Abstract

Kinesins play important roles in the progression and development of cancer. Kinesin family member C1 (KIFC1), a minus end-directed motor protein, is a novel Kinesin involved in the clustering of excess centrosomes found in cancer cells. Recently KIFC1 has shown to play a role in the progression of many different cancers, however, the involvement of KIFC1 in the progression of prostate cancer (PCa) is still not well understood. This study investigated the expression and clinical significance of KIFC1 in PCa by utilizing multiple publicly available datasets to analyze KIFC1 expression in patient samples. High KIFC1 expression was found to be associated with high Gleason score, high tumor stage, metastatic lesions, high ploidy levels, and lower recurrence-free survival. These results reveal that high KIFC1 levels are associated with a poor prognosis for PCa patients and could act as a prognostic indicator for PCa patients as well.

## Introduction

Prostate cancer (PCa) is the most commonly diagnosed cancer (aside from non-melanoma skin cancer) in men worldwide and the second leading cause of cancer deaths in American men [8]. In 2018 alone there were over 1.2 million new cases of PCa and over 300,000 deaths from PCa in the world [8]. The incidence rates and mortality rates of PCa are strongly correlated with age, with the highest incidence rates being in elderly men above 65 years of age [9]. While localized disease has a 5-year survival rate of 100%, patients that progress to metastatic disease have a five-year survival rate of only 30% [10]. Despite many therapeutic advances, metastatic prostate cancer remains incurable.

When stressed, cancer cells can undergo endoreplication, failed cytokinesis, or fusion which generates excess centrosomes within the cell. These same mechanisms also generate higher levels of genomic content in cancer cells taking cancer cells from aneuploid to polyploid or poly-aneuploid [11]. It is currently hypothesized that cells with elevated genomic content, e.g., polyploid giant cancer cells or poly-aneuploid cancer cells, may have advantages for surviving in stressful environments [12–18]. In animal cells there is a centrosome located at each spindle pole and each centrosome will be delivered to a single daughter cell during cell division and then eventually duplicate in the daughter cells S phase [19]. The correct distribution of centrosome number is critical for normal functioning of centrosome signaling and normal cell division [20]. In healthy mammalian cells, centrosome regulation is strictly maintained, however, centrosome aberrations are commonly observed in tumors [19]. Centrosome amplification can predispose cells to chromosomal instability (CIN) [21] and can also generate a multi-polar instead of a bi-polar spindle [22]. Multi-polarity is normally lethal but cells are able to avoid death by clustering their centrosomes and inducing bi-polar division.

Centrosome clustering in cancer cells is mediated by Kinesin family member C1 (KIFC1) (common paralogs: HSET, KIFC3, KIFC2, KIF14). KIFC1 is a minus end-directed motor protein involved in spindle pole organization and formation [23] and the clustering of excess centrosomes found in cancer cells [24, 25]. KIFC1 allows cancer cells with multiple centrosomes to survive cell division. KIFC1 is non-essential for normal somatic cells but necessary for the proper division of cancer cells with excess centrosomes [26, 27]. High KIFC1 levels have been reported in various cancer types such as breast cancer [28], hepatocellular carcinoma [29], and ovarian cancer [30]. Recently, KIFC1 has shown to be an important factor in prostate cancer progression and drug resistance by inhibiting cell death and conferring docetaxel resistance [24].

In this study, we demonstrate that high KIFC1 expression is associated with high grade, high stage, high ploidy, and metastatic PCa. Additionally, high KIFC1 levels are associated with poor recurrence-free survival in PCa as well as other cancer types.

## Methods

The Cancer Genome Atlas Prostate Adenocarcinoma (TCGA-PRAD) Cell 2015 [1], TCGA-PRAD provisional (accessed Nov 20, 2019), German Cancer Center (DKFZ) Cancer Cell 2018 [2], and Memorial Sloan Kettering Cancer Center (MSKCC) Cancer Cell 2010 [3] datasets were accessed via cBioPortal [4, 5]. The TCGA-PRAD provisional dataset was also accessed using UCSC Xena browser [6]. The TCGA-PRAD provisional dataset was used to analyze Gleason score and tumor stage, and compare PCa primary tissue with matched solid (normal) tissue. The TCGA-PRAD, Cell 2015 [1] dataset was used to analyze absolute doubling events and absolute extract ploidy. The DKFZ [2] dataset was used to analyze Gleason score and tumor stage. The MSKCC [3] dataset was used to analyze KIFC1 expression in primary and metastatic PCa and Gleason score. The MSKCC [3] dataset was also used to assess high/low KIFC1 expression and recurrence-free survival. GEPIA [7] was used to generate Kaplan Meier curves of KIFC1 expression and recurrence-free survival in TCGA-PRAD as well as thirty-three different TCGA datasets of different cancer types.

### Cell culture

PC3 and DU145 cells obtained from ATCC (PC3 cells were modified at the University of Michigan), were cultured in RPMI medium plus 10% FBS (VWR, product number: 97068–085) and 1% penicillin streptomycin. All cells were cultured in an incubator at 37 °C with 5% CO2. All cells underwent regular mycoplasma testing and STR profiling. Poly-aneuploid cancer cells (PACCs) were generated by treating PC3 or DU145 cells with 5 nM docetaxel for 72 h. PACCs were then isolated from the mixed population by filtration (pluriStrainer 15 μm cell strainer, SKU 43–50,015-03).

### Imaging

Phase-contrast images were taken of control PC3 cells and PC3 PACCs on an EVOS M7000 imaging system.

### Flow cytometry

Control PC3 cells and PC3 PACCs were stained with FxCycle PI/RNase Staining Solution (ThermoFisher Scientific cat# F10797). Both samples were run on a Bio-Rad S3E cell sorter and analyzed using FlowJo.

### Immunoblotting

Cells were lysed in radioimmunoprecipitation assay (RIPA) lysis buffer supplemented with Halt Protease and Phosphatase Single-Use Inhibitor cocktail (100x) (REF 78,442). Protein was fractionated on a 4–20% TGX gel and transferred to a nitrocellulose membrane. Membranes were incubated with anti-KIFC1 antibody (Abcam, Cat# ab172620, RRID: AB_2827938; 1:10,000) and anti-ß-Actin antibody (Sigma-Aldrich Cat# A5441, RRID: AB_476744; 1:10,000). Anti-rabbit and anti-mouse secondary were used for detection on a LI-COR Odyssey.

### Densitometry

Fiji software was used for densitometric analysis of the western blots. Resulting graphs are representative of three separate western blots.

### Statistical analysis

Statistical analysis was performed using GraphPad’s Prism software (GraphPad Software, San Diego, CA). One-way ANOVA or unpaired t-tests were performed to determine P values and significance across or between samples.

## Results

### High KIFC1 levels are associated with higher Gleason score

**Fig. 1:**
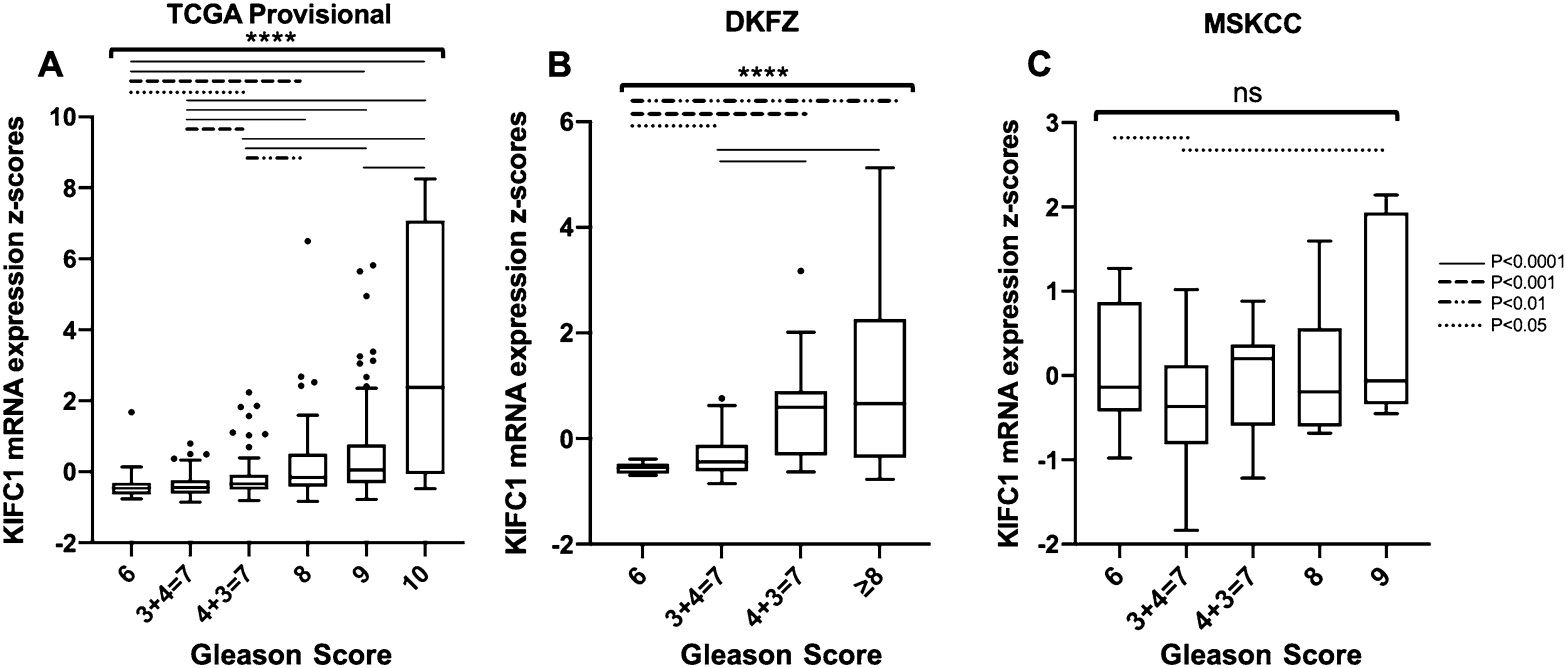
KIFC1 mRNA expression and Gleason score. **a** TCGA-PRAD provisional data (accessed on November 20, 2019) of KIFC1 mRNA expression and Gleason scores. (Gleason score: 6, *n* = 45; 3 + 4 = 7, *n* = 153; 4 + 3 = 7, *n* = 101; 8, *n* = 57; 9, *n* = 138; 10, *n* = 4). **b** DKFZ data of KIFC1 mRNA expression and Gleason scores (Gleason score: 6, *n* = 13; 3 + 4 = 7, *n* = 69; 4 + 3 = 7, *n* = 18; ≥ 8, *n* = 18). **c** MSKCC data of KIFC1 mRNA expression and Gleason scores (Gleason score: 6, *n* = 41; 3 + 4 = 7, *n* = 53; 4 + 3 = 7, *n* = 23; 8, *n* = 10; 9, *n* = 11. *P* values determined by one-way ANOVA or unpaired t-test. ns, *P* > 0.05; ****P ≤ 0.0001

KIFC1 mRNA expression was assessed in PCa patients with various Gleason scores. KIFC1 mRNA expression was increased with higher Gleason scores in three separate PCa datasets (Fig. 1). Both the TCGA-PRAD provisional (accessed November 20, 2019) and DKFZ [2] datasets demonstrated KIFC1 mRNA expression significantly increasing with higher Gleason score (*P* < 0.0001) (Fig. 1a, b). The MSKCC [3] dataset demonstrated a trend of KIFC1 mRNA expression increasing with higher Gleason score (*P* = 0.0584) (Fig. 1c). Patients with higher Gleason scores had higher KIFC1 mRNA expression indicating these patient samples most likely had higher levels of centrosome clustering mediated by KIFC1.

### KIFC1 expression may increase with PCa stage

**Fig. 2:**
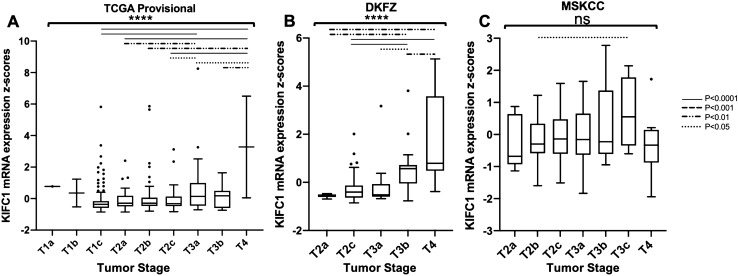
KIFC1 mRNA expression and Tumor Stage. **a** TCGA-PRAD provisional (accessed on November 20, 2019) data of KIFC1 expression and tumor stage (T1a, *n* = 1; T1b, *n* = 2; T1c, *n* = 174; T2a, *n* = 56; T2b, *n* = 55; T2c, *n* = 50; T3a, *n* = 36; T3b, *n* = 17; T4, *n* = 2). **b** DKFZ data of KIFC1 expression and tumor stage (T2a, *n* = 8; T2c, *n* = 66; T3a, *n* = 16; T3b, *n* = 19; T4, *n* = 7). **c** MSKCC data of KIFC1 expression and tumor stage (T2a, *n* = 9; T2b, *n* = 48: T2c, *n* = 29; T3a, *n* = 30; T3b, *n* = 13; T3c, *n* = 4; T4, *n* = 8). *P* values determined by one-way ANOVA or unpaired t-test. ns, *P* > 0.05; ****P ≤ 0.0001

Across the 393 patient samples assessed in the TCGA-PRAD provisional dataset (accessed November 20, 2019) KIFC1 mRNA expression was significantly increased with higher tumor stage (*P* < 0.0001) (Fig. 2a). Similar trends were shown across the 116 patient samples in the DKFZ [2] dataset where KIFC1 mRNA expression was also significantly increased with higher tumor stage (*P* < 0.0001) (Fig. 2b). Data from the 141 patient samples in the MSKCC [3] dataset demonstrated KIFC1 expression was highest in tumor stage T3C but did not significantly increase with higher tumor stage (*P* = 0.2879) (Fig. 2c). KIFC1 mRNA expression increased as tumor stage increased in multiple datasets indicating higher stage tumors likely have higher levels of KIFC1 mediated centrosome clustering.

### KIFC1 expression is highest in metastatic PCa

**Fig. 3:**
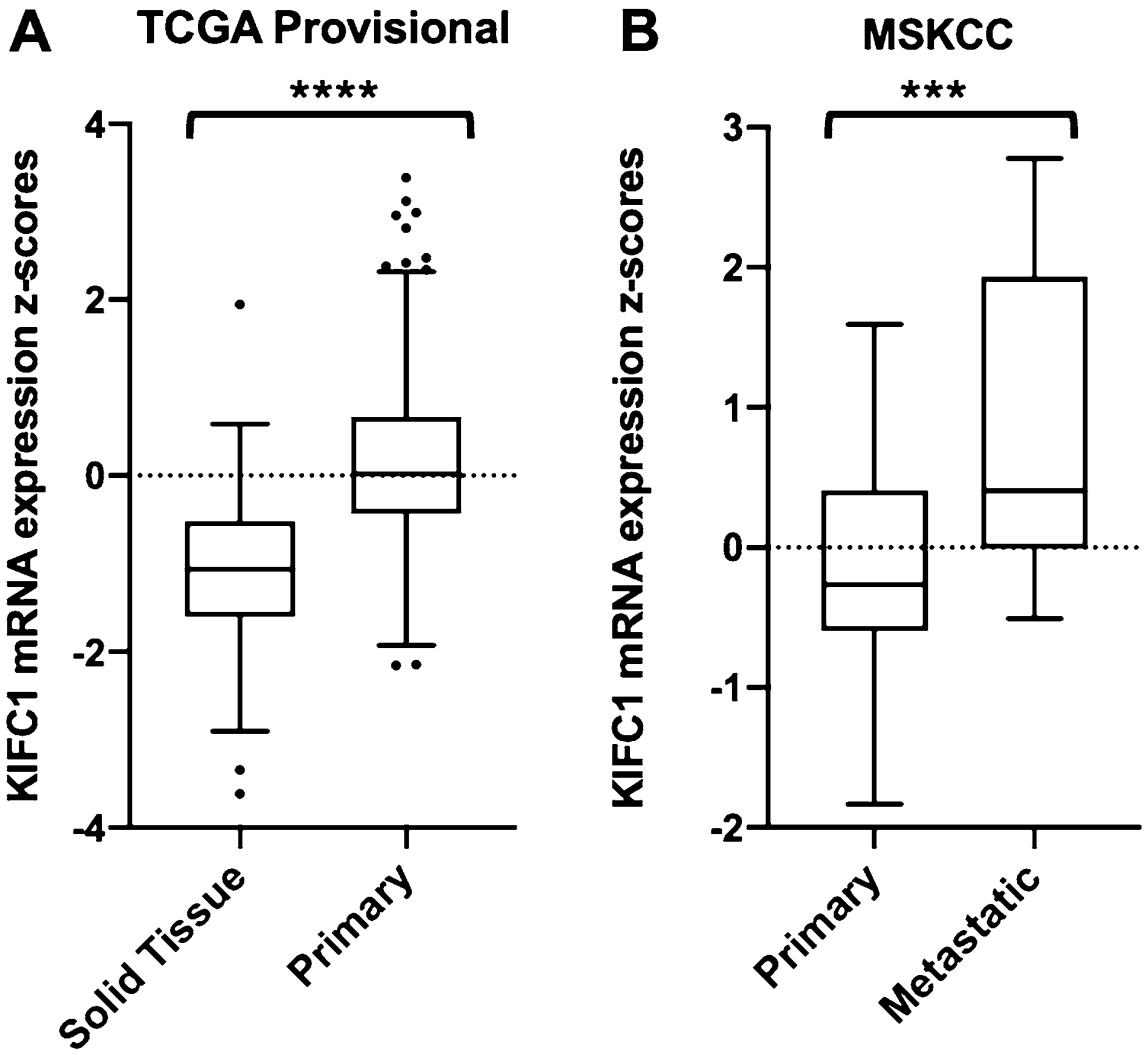
KIFC1 mRNA expression and metastatic disease. **a** TCGA-PRAD provisional data comparing KIFC1 mRNA expression in matched solid (normal) tissue (*n* = 52) and primary PCa (*n* = 479). **b** MSKCC data comparing primary (*n* = 130) and metastatic PCa (*n* = 9) to KIFC1 mRNA expression. *P* values determined by unpaired t-test. ***P ≤ 0.001; ****P ≤ 0.0001

KIFC1 mRNA expression was higher in metastatic PCa lesions than in the primary tumors. In TCGA-PRAD provisional dataset primary tumor KIFC1 mRNA expression was higher than matched solid tissue (Fig. 3a). The MSKCC [3] dataset showed KIFC1 mRNA expression was higher in metastatic lesions than in the primary tumor (Fig. 3b). KIFC1 expression was higher in primary tumors than in matched solid (normal) tissue as well as higher in metastatic lesions than in the primary tumor indicating that metastatic PCa lesions may require higher levels of centrosome clustering mediated by KIFC1.

### High KIFC1 expression is associated with lower recurrence-free survival in PCa as well as other cancer types

**Fig. 4:**
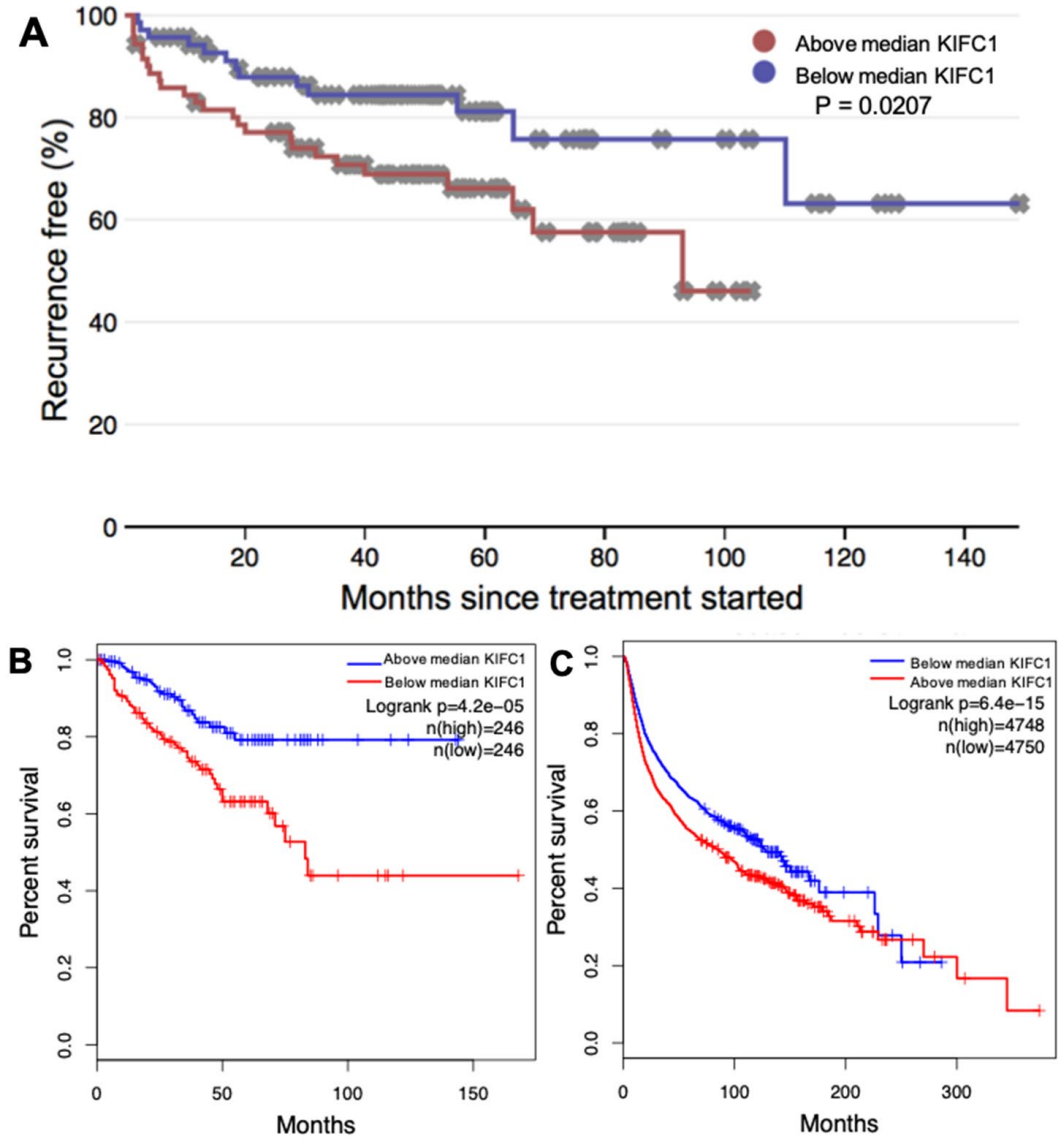
High KIFC1 expression is correlated with lower recurrence-free survival in PCa and other cancer types. Kaplan Meier curves and P values generated from betastasis.com and GEPIA comparing KIFC1 expression and recurrence-free survival. **a** Kaplan Meier curve from betastasis.com utilizing the MSKCC dataset comparing above/below median KIFC1 mRNA expression in PCa (*n*(above median) = 70; *n*(below median) = 71) (*P* = 0.0207). **b** Kaplan Meier curve generated from GEPIA of the TCGA-PRAD dataset comparing above/below median KIFC1 expression (*n*(above median) = 246; *n*(below median) = 246) (*P* = 4.2E-05). **c** Kaplan Meier curve generated from GEPIA of the thirty-three compiled TCGA datasets available comparing above/below median KIFC1 expression and recurrence-free survival across multiple cancer types (*n*(above median) = 4748; *n*(below median) = 4750) (*P* = 6.4e-15)

This study compared recurrence-free survival between patients with high and low KIFC1 mRNA expression levels. Recurrence-free survival in PCa describes time to biochemical recurrence (BCR) which refers to a rise in the blood level of prostate-specific antigen (PSA) in PCa patients after initial treatment. In the MSKCC [3] PCa dataset patients with higher than median KIFC1 mRNA expression levels had lower recurrence-free survival (*P* = 0.0207) (screenshots taken directly from bestasatsis.com) (Fig. 4a).

We also used GEPIA [7] to generate Kaplan Meier curves using data from TCGA-PRAD dataset. TCGA-PRAD patients with above-median KIFC1 expression levels had lower recurrence-free survival compared to patients with below median KIFC1 expression (*P* = 4.2E-05) (Fig. 4b). Next, we looked at KIFC1 expression across multiple cancer types. Thirty-three TCGA datasets available on GEPIA [7] (including TCGA-PRAD) were analyzed for KIFC1 expression. Patients with above-median KIFC1 expression had lower recurrence-free survival when assessing all of the cancers at once (Fig. 4c) (*P* = 6.4E-15). Additionally, when analyzed separately, eleven of the thirty-three datasets analyzed had significant differences (*P* < 0.05) when comparing median high to low KIFC1 mRNA expression: adrenocortical carcinoma (ACC), kidney chromophobe (KICH), kidney renal clear cell carcinoma (KIRC), kidney renal papillary cell carcinoma (KIRP), brain lower grade glioma (LGG), liver hepatocellular carcinoma (LIHC), mesothelioma (MESO), prostate adenocarcinoma (PRAD), sarcoma (SARC), thyroid carcinoma (THCA), and uveal melanoma (UVM). Overall, high KIFC1 mRNA expression was associated with lower recurrence-free survival in PCa as well as other cancer types, indicating that patients with these cancer types and high KIFC1 levels have a poorer prognosis.

### High KIFC1 expression is associated with higher ploidy levels

**Fig. 5:**
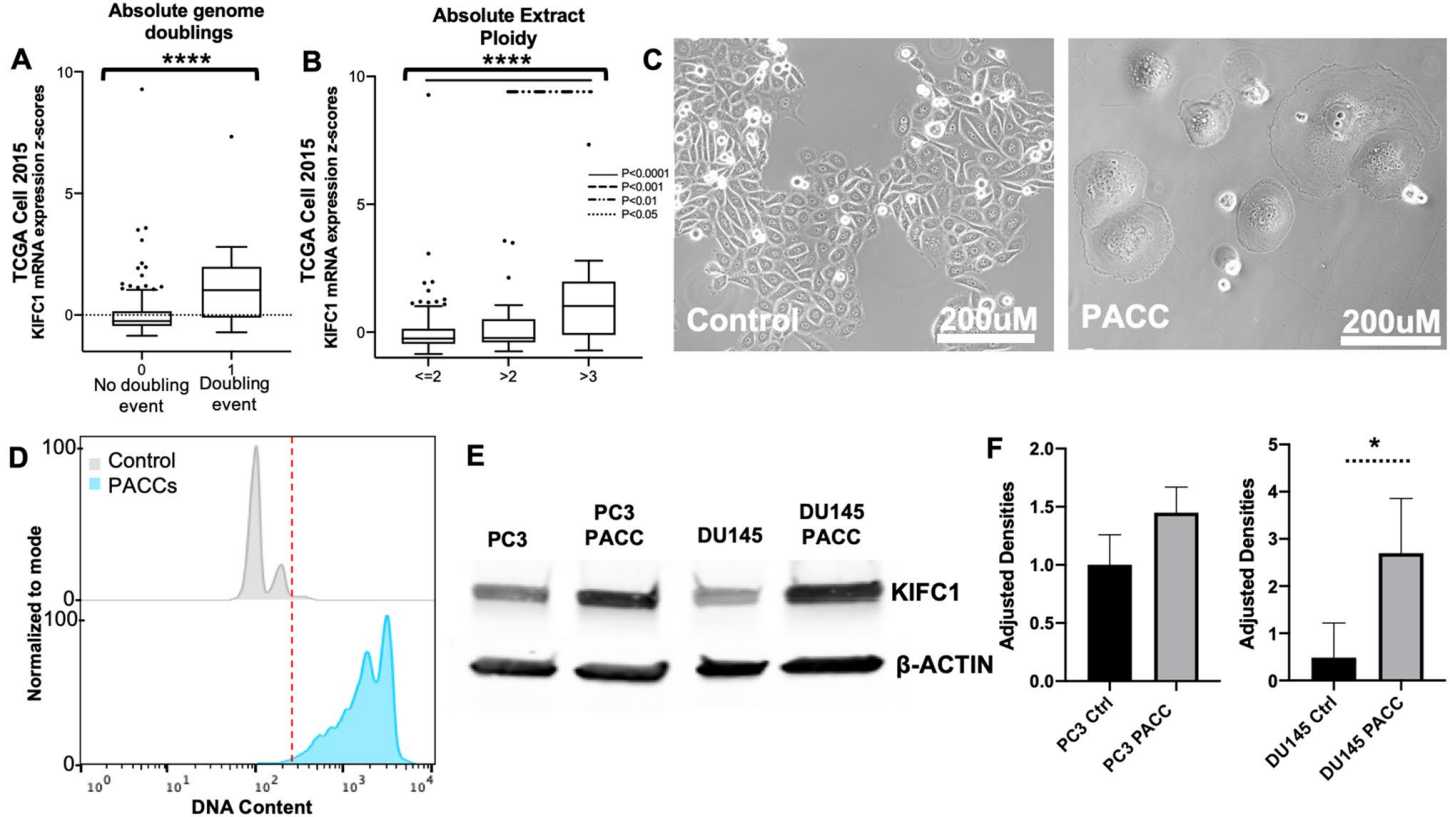
Relationship of KIFC1 expression and ploidy levels. **a** TCGA-PRAD Cell 2015 data comparing KIFC1 mRNA expression and absolute genome doublings (Without a doubled genome, *n* = 229; With a doubled genome, *n* = 18). **b** TCGA-PRAD Cell 2015 data comparing KIFC1 mRNA expression and absolute extract ploidy (< = 2, *n* = 192; > 2, *n* = 37; > 3, *n* = 18). **c** Phase-contrast images of control PC3 cells and PC3 PACCs. **d** Flow cytometry analysis of DNA content in control PC3 cells and PC3 PACCs after filtration enrichment (all cells to the right of the red line are > 4N). **e** Western blot comparing KIFC1 expression in PC3 cells, PC3 PACCs, DU145 cells, and DU145 PACCs (results are representative of three separate experiments). **f** Densitometric analysis of western blots. *P* values determined by one-way ANOVA or unpaired t-test. *P ≤ 0.05; ****P ≤ 0.0001

In TCGA-PRAD, Cell 2015 [1], dataset KIFC1 mRNA expression was analyzed for absolute genome doublings and absolute extract ploidy. Absolute genome doublings and absolute extract ploidy were determined using the ABSOLUTE algorithm [31] which deduces malignant cell ploidy directly from analysis of somatic DNA alterations. KIFC1 mRNA expression was significantly increased in samples with absolute genome doublings (*P* < 0.0001) (Fig. 5a). KIFC1 expression was also significantly higher in samples with higher absolute extract ploidy (Fig. 5b) (*P* < 0.0001).

To directly assess ploidy in cancer cells, we generated and isolated poly-aneuploid cancer cells (PACCs) from two different cell lines. PACCs are cancer cells containing a doubled (or greater multiple, e.g., 4 N, 8 N…) aneuploid genome, also referred to as polyploid giant cancer cells (PGCCs) or multi-nucleated cancer cells [11–18]. PACCs were generated by treating cells with 5 nM docetaxel for 72 h. They were then isolated from the population using a 15um filter. PACCs were visualized by phase-contrast imaging (Fig. 5c). Additionally, PACC ploidy was verified by the assessment of DNA content in all cells by flow cytometry (Fig. 5d). Utilizing western blotting we demonstrated that KIFC1 protein levels were higher in PACCs than their parental cell lines (Fig. 5e). We also demonstrated this by quantifying three separate western blots via densitometric analysis (Fig. 5f). This data demonstrated higher ploidy samples are associated with high KIFC1 mRNA expression and high KIFC1 protein levels. This could indicate that higher ploidy cancers need higher levels of centrosome clustering mediated by KIFC1.

## Discussion

Genomic instability, a hallmark of cancer, facilitates the acquisition of mutations that can drive cancer evolution and drug resistance. Aneuploidy, the presence of an abnormal number of chromosomes in a cell, is a characteristic of genomic instability and introduces more genetic variation, allowing cells to adapt in changing or stressful environments [21]. Aneuploid cells that undergo endoreplication, failed cytokinesis, or fusion become polyploid/poly-aneuploid cancer cells (cells containing more than two paired sets of chromosomes) and contain excess centrosomes. Excess centrosomes can lead to cell death when a cell attempts multi-polar division. However, centrosomes can be clustered to promote bi-polar division and cell survival. KIFC1 is an important kinesin for cancer cells that clusters excess centrosomes to allow for the successful division and survival of cancer cells. KIFC1, while not expressed in somatic cells, is widely expressed in cancers such as ovarian [30], breast [28], bladder [32], lung [33], kidney [34], and we report here, prostate. Elevated levels of KIFC1 have been shown to confer drug resistance in breast cancer [35] as well as prostate cancer [24]. KIFC1 has recently been described as important to PCa progression [24].

The Gleason scoring system and the TNM staging system are utilized to categorize prostate cancer aggressiveness and disease extent. The Gleason scoring system is one of the most common grading systems used for PCa [10] and Gleason scores have been shown to be powerful indicators of PCa recurrence and mortality [36]. We report here that patients with higher Gleason scores have higher levels of KIFC1 mRNA expression suggesting the capacity for higher levels of KIFC1 mediated centrosome clustering in those patients’ tumors. The DKFZ [2] and TCGA-PRAD provisional (accessed Nov. 20, 2019) datasets demonstrated significant increases in KIFC1 mRNA expression with higher T stages in primary tumors. While the MSKCC dataset did not show significant differences, it did show a trend of increasing KIFC1 mRNA expression and higher T stages and Gleason scores. These findings that KIFC1 mRNA expression increases as tumor stage progresses in multiple datasets indicates that higher stage tumors may require higher levels of KIFC1 mediated centrosome clustering.

Further investigation of KIFC1 mRNA expression in clinical datasets led to the evaluation of KIFC1 mRNA expression in metastatic lesions. In this study, KIFC1 mRNA expression was found to be higher in metastatic PCa lesions compared to the primary tumor. It was also shown that higher KIFC1 levels are present in primary tumor samples compared to matched solid (normal) tissue samples. These results reveal that higher levels of centrosome clustering, mediated by KIFC1, are present in metastatic lesions compared to the primary tumor or normal tissue.

KIFC1 expression not only increased with higher Gleason scores, tumor stages, and metastatic disease; high KIFC1 expression was also associated with lower recurrence-free survival in PCa patients as well as patients with other cancer types. A significant difference was shown between the high/low KIFC1 expression groups in PCa. Additionally, thirty-three TCGA datasets of other cancer types, when analyzed simultaneously, demonstrated a trend with higher KIFC1 expression and lower recurrence-free survival. This data delineates KIFC1 as a potential prognostic factor in PCa and also demonstrates that high KIFC1 mRNA expression is not only associated with a poor prognosis in PCa, but in many other cancer types as well.

KIFC1 mRNA expression was higher in samples with absolute genome doublings and higher absolute extract ploidy. In addition to KIFC1 mRNA expression, this study also observed KIFC1 protein levels in higher ploidy cells (PACCs). PACCs are large atypical cancer cells with multiple copies of DNA [15]. PACCs have been documented in several different baseline cancer lines including ovarian cancer cell lines HEY and SKOv3, and breast cancer cell line MDA-MB-231 [15]. Further investigation of PACCs showed that KIFC1 protein levels were higher in PCa PC3 PACCs and PCa DU145 PACCs compared to control PC3 and DU145 cells. Overall, as ploidy levels increased so did KIFC1 levels demonstrating that higher ploidy cells contain higher levels of KIFC1.

**Fig. 6:**
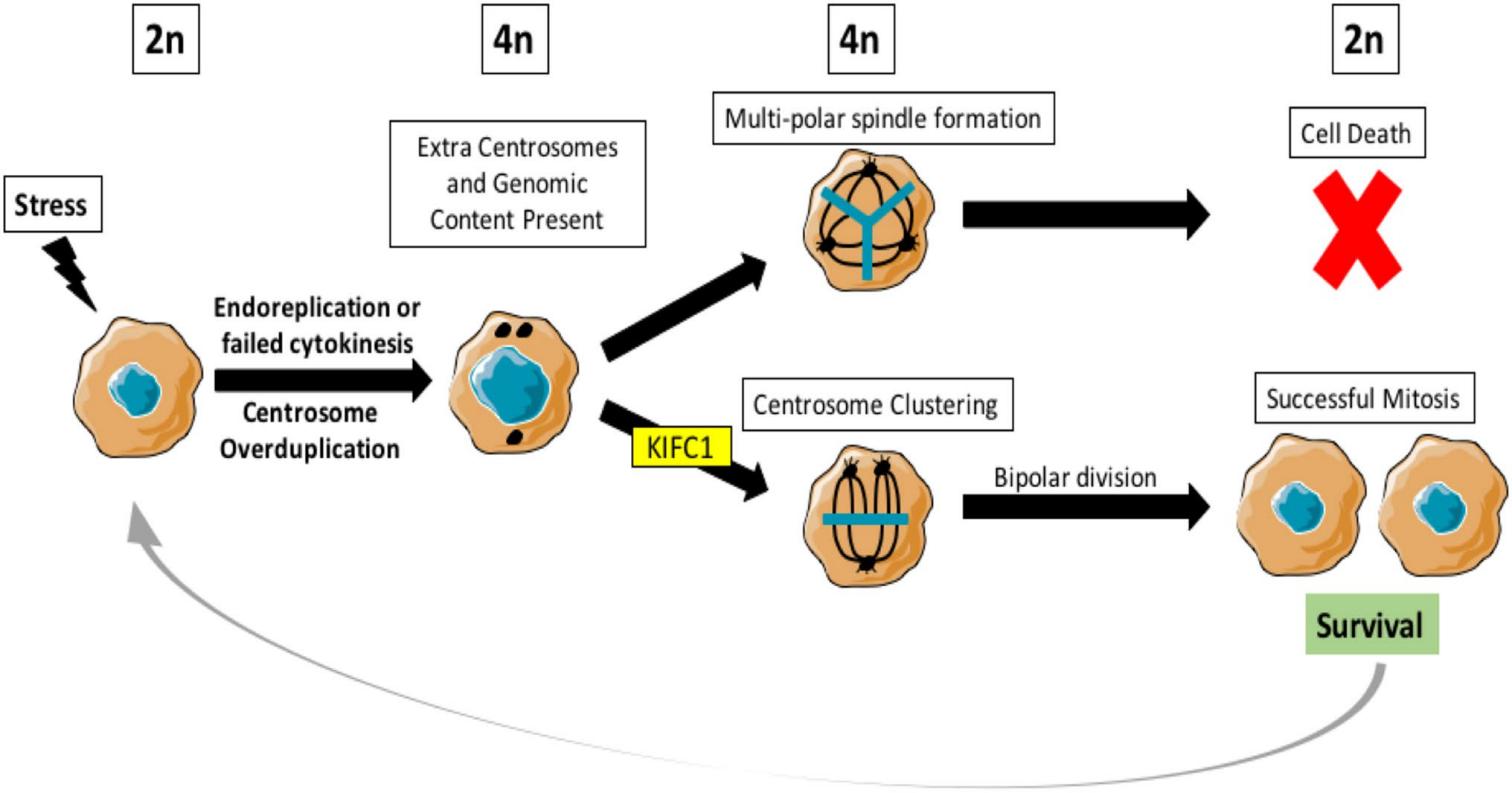
KIFC1 allows cancer cell survival through centrosome clustering. When stressed, cells may undergo endoreplication, failed cytokinesis, or fusion causing an over duplication of centrosomes and an increase in genomic content. Cells with over-duplicated centrosomes may attempt to divide unevenly and form a multipolar spindle. KIFC1 is capable of taking cells with multipolar spindles and clustering their centrosomes allowing for bipolar division. This bipolar division allows cell survival and continued proliferation

Our study promotes a model for KIFC1 as a novel kinesin in PACC and other high ploidy cell survival. Cancer cells that undergo endoreplication, failed cytokinesis, or fusion contain over-duplicated centrosomes and higher levels of genomic content. During division, a multi-polar spindle can form in response to the presence of excess centrosomes. Microtubules then bind incorrectly and genomic content is distributed unevenly between the multiple poles. This ultimately results in either (1) A cell not progressing past the mitotic checkpoint or (2) Death from the inability to transfer the necessary amount of genomic material to each daughter cell. KIFC1 is a novel kinesin that clusters excess centrosomes in cancer cells allowing the formation of bi-polar spindles, normal division, and cell survival (Fig. 6).

This study presents evidence of high KIFC1 expression in PCa indicating a poorer prognosis. High KIFC1 expression was correlated with high Gleason score, high tumor stage, metastatic lesions, lower recurrence-free survival, and higher ploidy levels. This is also the first evidence that showed PACCs had higher KIFC1 protein levels than normal parental cells. This information leads us to hypothesize if KIFC1 could be inhibited effectively in combination with other treatment options it would lead to increased cancer cell lethality.

## Data Availability

The data that supports the findings of this study are available in The Cancer Genome Atlas Prostate Adenocarcinoma (TCGA-PRAD) Cell 2015 [1], TCGA-PRAD Provisional (accessed Nov 20, 2019), German Cancer Center (DKFZ) Cancer Cell 2018 [2], and Memorial Sloan Kettering Cancer Center (MSKCC) Cancer Cell 2010 [3] datasets. Data were derived from the following databases available in the public domain: cBioPortal [4, 5], UCSC Xena browser [6], betastasis.com, and GEPIA [7].

## References

[1] Abeshouse A (2015). The Molecular Taxonomy of Primary Prostate Cancer. Cell.

[2] Gerhauser C (2018). Molecular Evolution of Early-Onset Prostate Cancer Identifies Molecular Risk Markers and Clinical Trajectories. Cancer Cell.

[3] Taylor BS (2010). Integrative Genomic Profiling of Human Prostate Cancer. Cancer Cell.

[4] Gao J (2013). Integrative Analysis of Complex Cancer Genomics and Clinical Profiles Using the cBioPortal. Sci. Signal..

[5] Cerami E (2012). The cBio Cancer Genomics Portal: An Open Platform for Exploring Multidimensional Cancer Genomics Data. Cancer Discov.

[6] Goldman MJ (2020). Visualizing and interpreting cancer genomics data via the Xena platform. Nat Biotechnol.

[7] Tang Z, Li C, Kang B, Gao G, Li C, Zhang Z (2017). GEPIA: a web server for cancer and normal gene expression profiling and interactive analyses. Nucleic Acids Res.

[8] Bray F, Ferlay J, Soerjomataram I, Siegel RL, Torre LA, Jemal A (2018). Global cancer statistics 2018: GLOBOCAN estimates of incidence and mortality worldwide for 36 cancers in 185 countries”. CA Cancer J Clin.

[9] Rawla P (2019). Epidemiology of Prostate Cancer. World Journal of Oncology.

[10] “Prostate Cancer - Statistics,” *Cancer.Net*, Jun. 25, 2012. https://www.cancer.net/cancer-types/prostate-cancer/statistics (accessed Feb. 04, 2020).

[11] Mosieniak G, Sikora E (2010). Polyploidy: the link between senescence and cancer. Curr Pharm Des.

[12] Erenpreisa J (2008). Endopolyploidy in irradiated p53-deficient tumour cell lines: Persistence of cell division activity in giant cells expressing Aurora-B kinase. Cell Biol Int.

[13] Fei F (2015). The number of polyploid giant cancer cells and epithelial-mesenchymal transition-related proteins are associated with invasion and metastasis in human breast cancer. J Exp Clin Cancer Res.

[14] Chen J (2019). Polyploid Giant Cancer Cells (PGCCs): The Evil Roots of Cancer. Curr Cancer Drug Targets.

[15] Zhang S, Mercado-Uribe I, Xing Z, Sun B, Kuang J, Liu J (2014). Generation of cancer stem-like cells through the formation of polyploid giant cancer cells. Oncogene.

[16] Erenpreisa JA, Cragg MS, Fringes B, Sharakhov I, Illidge TM (2000). Release of Mitotic Descendants by Giant Cells from Irradiated Burkitt’s Lymphoma Cell Lines. Cell Biol Int.

[17] Zhang L, Wu C, Hoffman RM (2015). Prostate Cancer Heterogeneous High-Metastatic Multi-Organ-Colonizing Chemo-Resistant Variants Selected by Serial Metastatic Passage in Nude Mice Are Highly Enriched for Multinucleate Giant Cells. PLoS ONE.

[18] Zheng L (2012). Polyploid cells rewire DNA damage response networks to overcome replication stress-induced barriers for tumour progression. Nat Commun.

[19] LoMastro GM, Holland AJ (2019). The Emerging Link between Centrosome Aberrations and Metastasis. Dev Cell.

[20] Nigg EA, Raff JW (2009). Centrioles, Centrosomes, and Cilia in Health and Disease. Cell.

[21] Giam M, Rancati G (2015). Aneuploidy and chromosomal instability in cancer: a jackpot to chaos. Cell Div.

[22] Vitre BD, Cleveland DW (2012). Centrosomes, chromosome instability (CIN) and aneuploidy. Curr Opin Cell Biol.

[23] Walczak CE, Vernos I, Mitchison TJ, Karsenti E, Heald R (1998). A model for the proposed roles of different microtubule-based motor proteins in establishing spindle bipolarity. Curr Biol.

[24] Sekino Y (2017). KIFC1 induces resistance to docetaxel and is associated with survival of patients with prostate cancer. Urologic Oncology: Seminars and Original Investigations.

[25] Xiao Y-X, Yang W-X (2016). KIFC1: a promising chemotherapy target for cancer treatment?. Oncotarget.

[26] Rath O, Kozielski F (2012). Kinesins and cancer. Nat Rev Cancer.

[27] Kwon M (2008). Mechanisms to suppress multipolar divisions in cancer cells with extra centrosomes. Genes Dev.

[28] Pannu V (2015). HSET overexpression fuels tumor progression via centrosome clustering-independent mechanisms in breast cancer patients. Oncotarget.

[29] Fu X (2018). KIFC1, a novel potential prognostic factor and therapeutic target in hepatocellular carcinoma. Int J Oncol.

[30] Pawar S (2014). KIFCI, a novel putative prognostic biomarker for ovarian adenocarcinomas: delineating protein interaction networks and signaling circuitries. Journal of Ovarian Research.

[31] Carter SL (2012). Absolute quantification of somatic DNA alterations in human cancer. Nat Biotechnol.

[32] Boris A (2014). Mp22-18 identification of novel gene expression markers for bladder cancer diagnostics. J Urol.

[33] Grinberg-Rashi H (2009). The Expression of Three Genes in Primary Non-Small Cell Lung Cancer Is Associated with Metastatic Spread to the Brain. Clin Cancer Res.

[34] Chan JY (2011). A Clinical Overview of Centrosome Amplification in Human Cancers. Int J Biol Sci.

[35] Martin SK, Kyprianou N, Fisher PB, Tew KD (2015). Chapter Three-Exploitation of the Androgen Receptor to Overcome Taxane Resistance in Advanced Prostate Cancer. Advances in Cancer Research.

[36] Varma M, Berney D, Oxley J, Trpkov K (2018). Gleason score assignment is the sole responsibility of the pathologist. Histopathology.

